# Disclosure of Conflict of Interest at a Professional Surgical Society Annual Meeting

**DOI:** 10.7759/cureus.98417

**Published:** 2025-12-03

**Authors:** Brij R Chhabra, Atul K Madan, David S Tichansky

**Affiliations:** 1 Surgery, Southern California Bariatrics, Los Angeles, USA; 2 Surgery, University of California Los Angeles David Geffen School of Medicine, Los Angeles, USA

**Keywords:** conflict of interest, disclosure, industry payment, research, scientific presentations

## Abstract

Introduction

Relationships with industry may create a potential conflict of interest (COI). Disclosure of COI is essential for integrity of educational programming. In 2013, Center for Medicare and Medicaid Services (CMS) developed the OpenPayments™ database to facilitate reporting, visibility, and accountability for US physicians. While the Society of American Gastrointestinal and Endoscopic Surgeons (SAGES) and the Accreditation Council for Continuing Medical Education (ACCME) mandate disclosing relationships with industry, we sought to investigate only significant potential COIs. We hypothesize that most authors disclose significant potential COIs appropriately and those that do not disclose only have minor potential COI.

Methods

Payment information for authors at the 2023 SAGES meeting was extracted from the OpenPayments™ database (https://openpaymentsdata.cms.gov). Authors not initially found were additionally searched via Google, LinkedIn, affiliation websites, and/or state medical board websites. National Provider Identifier (NPI) numbers were utilized to verify identities as needed. We defined significant potential COI as total payments > $10,000 for two years prior to the meeting. Authors were divided as disclosing COI (Disclosed) versus not disclosing COI (Not Disclosed), the latter including those with no mention of COI, stated “no relevant COI”, or stated “no COI.”

Results

There were 792 authors in 2023 and 296 eligible authors. The proportion of disclosed authors in 2023 was 16% (47/296); the incidence of significant potential COI was 25% (73/296). The average potential COI payment received was $104,284 but did not statistically differ between the Disclosed vs. Not Disclosed groups; however, only 49% (36/74) of authors that had potential COI were actually in the Disclosed group. The highest paid author in the Not Disclosed group received over $868,000.

Conclusion

The majority of authors appropriately disclosed potential COIs at the 2023 SAGES meeting. Contrary to our hypothesis, Not Disclosed authors may still have potential COIs. Disclosing the mere existence of a relationship seems insufficient as some authors received large sums of money. All presentations should include the dollar amounts for full disclosure of potential COI for future programs.

## Introduction

Bias continues to infiltrate every channel of information. Scientific literature and presentations at scientific meetings are not immune from the presence of information that may promote a personal interest as opposed to objective fact. Conflict of interest (COI) has been defined as occurring when “a primary interest tends to be unduly influenced by a secondary interest” [1]. While non-financial COIs do exist [2], particular attention has been given to the potential COI created in the financial sphere by direct payments to providers from companies that manufacture and supply healthcare products. Unfortunately, these payments are pervasive and have been historically underreported. Tian et al. found that as many as 92% of published studies in one sample, comprising 669 authors, had received undisclosed payments of over $250 [3]. It has also been shown that COIs have influence at the editorial level of medical journals [4] and at the leadership level at medical institutions [5].

But it is important to realize that financial COI does not always produce misinformation and conveyance of misinformation is not always malicious, or even conscious. In fact, research has shown lack of neutral objectivity when a COI exists. [6] In an effort to curtail the conscious (or even subconscious) influence of personal financial gain upon submission of scientific investigation to medical literature and scientific meetings, most scientific societies have put forth guidelines for disclosure of potential COI. In fact, Accreditation Council for Continuing Medical Education (ACCME) has even mandated this compliance in its accreditation process. ACCME defines ineligible companies as “those whose primary business is producing, marketing, selling, re-selling, or distributing healthcare products used by or on patients.” These potential COIs must be disclosed for all Continuing Medical Education (CME) activities.

The theory is to use transparency as a vehicle to reduce bias. The Society of Gastrointestinal and Endoscopic Surgeons (SAGES) has been at the forefront of mandating investigator disclosure and has a solid track-record of requiring disclosure and publishing the results of the same [7-10]. Early as the society rolled out disclosure policies, they noted a three-fold increase in presenter disclosures from 2011 to 2013 and this increase in disclosure was correlated with a decrease in perceived bias [7].

To augment transparency of direct-to-provider industry payments, the Center for Medicare and Medicaid Services (CMS) created the Open Payments Database (OPD) in 2013, which tracks the mandatory reporting of payment amounts by industry to providers and healthcare organizations. Armed with objective dollar amounts, Lois et al. later demonstrated that while disclosure increased at SAGES meetings, significant under-reporting was occurring [8]. Additionally, they reported an association between the magnitude of payments and specific topics of research, implying that through many payments to a large number of providers industry could aggregate potential influence, yet this cumulative COI may go unnoticed at the individual COI disclosure level [8]. While the influence of SAGES on COI disclosure continued to increase on a year-on-year basis to greater than 90%, absolute transparency has remained elusive [9]. In the years to follow, in spite of their strong record of accomplishment, SAGES was cited for non-compliance with ACCME standards [10]. The society has since created additional tools, including an infographic, making decisions around disclosure simple and clear.

We hypothesize that most authors disclose potential COIs appropriately at SAGES and those that do not disclose only have minor potential COIs. We further investigate if binary disclosure of presence or absence of COI is insufficient and that full transparency of the amount of payments is necessary to give audiences the information needed to determine if bias exists. 

## Materials and methods

This cross-sectional study chose to include authors who gave oral presentations at the SAGES 2023 annual meeting. Plenary and scientific sessions were included; however, “quick shots”, video presentations, resident/fellow forum presentations, and non-CME presentations were not included in this study. The identity of all of the authors of each presentation was compiled, including names, organizations, and locations. The authors included were any and all those listed on the title slide. Using this data, the CMS OpenPaymentsData database (https://openpaymentsdata.cms.gov) was initially searched. As these data only includes physicians from the United States, other authors were not included in the analysis. In the same vein, students, research assistants, residents, fellows, and other non-physician researchers were not included in this study. The aggregate amount of payment given to each physician was recorded for the previous last two years. We arbitrarily defined significant potential COI as receiving higher than $10,000 for the last two years prior to the meeting.

Each presentation was reviewed to determine if each author had disclosed potential COI. All authors that were included in each presentation were included in this study. Two groups were created and defined as Disclosed and Not Disclosed. The Disclosed group disclosed a COI. The Not Disclosed group consisted of authors that (1) stated they had no COI to disclose, (2) stated they had no relevant COI disclosures, or (3) did not mention disclosures in their presentations.

Certain authors could not be found solely on the basis of their names and locations. For these authors, we implemented additional searches via Google, Linkedin, practice/institution affiliation, and state medical board website to determine their correct name and/or location. Additionally, National Provider Identifier (NPI) numbers were searched via Google for physicians who had common names and/or did not use their complete official name in the presentations. These NPI numbers were inputted into the OpenPaymentData website to ensure we had the correct physician.

Data were analyzed with two-tailed Mann-Whitney tests using https://astatsa.com/ statistical program (Navendu Vasavada, Mountain View, CA, USA under Creative Commons Attribution 4.0 international). An institutional review board exemption was received for this investigation.

## Results

There were 792 authors; however, a majority (496/792, 62%) of the authors were excluded based on the above exclusion criteria. A total of 296 authors were included in this study. There were 249 (84%) authors in the Not Disclosed group and 47 (16%) in the Disclosed group. Table 1 demonstrates that the number of significant potential COI in Disclosed vs. Not Disclosed authors. Only 37/296 (12.5%) of total authors had actual significant potential COI that they did not disclose. Interestingly, 11/296 (3.7%) disclosed that they had COI but did not reach our definition of significant potential COI.

**Table 1:** Number (Percentage) of significant potential COI in Disclosed vs. Not Disclosed authors

	COI	No COI	Total
Not Disclosed	37 / 249 (14.9%)	212 / 249 (85.1%)	249
Disclosed	36 / 47 (76.6%)	11 / 47 (23.4%)	47

**Figure 1:**
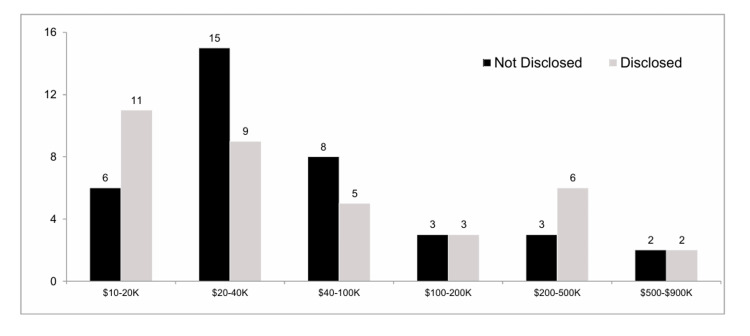
Distribution of the amount of COI by Disclosed vs. Not Disclosed authors Distribution of the amount of COI by Disclosed versus Not Disclosed authors. There was no overall statistical significant difference between the Disclosed and Not Disclosed authors.  Even at the highest level of dollar amount, there were authors from both groups.

The average payment for Disclosed authors who had significant potential COI was $116,447 versus $93,056 for the Not Disclosed authors (p=NS). The median payment for both groups was $30,415.18. Figure 1 demonstrates the distribution of the amount of COI by Disclosed vs. Not Disclosed authors. Over a quarter of the authors (19/73, 26%) that had significant potential COI received over $100,000. The highest paid author received over $868,000.

## Discussion

Disclosure of COI is essential for scientific societies and audiences alike. SAGES as an organization has been at the forefront of promoting and improving disclosure to decrease perceived and real bias in presented information. SAGES membership comprises surgical specialties and surgeons who, out of necessity, are heavy users of surgical devices and new technology. Presumably because of this fact, the society and its members are attractive development partners for industry, thus leading to potential payment for collaboration and consultative services. This creates a challenge for investigators because, even potentially at the subconscious level, judgment becomes biased when self-interest is involved [6,11-14].

There have been multiple investigations studying the interventions developed and mandated by SAGES [7-10]. Stain et al. examined the effect of establishing a Conflict of Interest Task Force. Through education and monitoring, they noted a 200% increase in disclosure practices from 2011 to 2013 and a 65% decrease in audience perceived bias in the presentations from 2010 to 2013 [7]. Fast forwarding to the era of the OPD, Lois et al. examined concordance between speaker-disclosed and OPD-reported payments greater than $500. This paper found disclosure to be high but concordance with the OPD showed under-reporting [8]. Perhaps most interesting from the same investigation was that robotics and hernia repair, as topics, accounted for over $5 million in payments (>80 % of the total for the meeting) with a single robot vendor contributing nearly $2 million. Through some combination of additional SAGES interventions and the Hawthorne effect, Lois et al. further reported increases in disclosure and concordance with the OPD reports as well as a decrease in industry payments by 44% [9].

This investigation takes a fresh look at concordance of self-disclosure with the OPD, with a focus on payment size for presenters at the 2023 SAGES meeting. Specifically, we defined significant potential COI of aggregate payments greater than $10,000. It should be stated that the majority of authors followed the disclosure rules at the 2023 SAGES Annual Meeting Scientific Sessions, aligned with previous SAGES meetings. To highlight those authors who disclosed or received payments, this study combines authors who disclosed “Nothing to disclose”, “No relevant disclosures”, and those who made no mention of disclosures. The term “no relevant disclosures” should not exist and be permitted. Those who document that do not understand the concept of disclosures and need to be educated. Contrary to our hypothesis, Not Disclosed authors may still have significant potential COI. Of note is that 26% of authors that received significant potential COI payments received more than $100,000 going up to over $800,000, regardless of disclosure. Behavioral psychology would infer that an unmitigable bias likely exists; however, these biases may be subconscious, which potentially compounds the issue.

A valid weakness of this study is that it likely understates the magnitude of potential significant COI because we did not look at and/or verify other COIs (owning stock options, owning stock, family members working in companies, etc.). While family member employers and stock options may not be considered potential COI by ACCME, we feel that these items sometimes may represent more of a COI than even direct payments. While ACCME has done a good job in setting guidelines, we feel that the authors should disclose appropriately on their own. In a sense, any presentation has some type of COI and/or bias. Presentations are an important part of advancement so presentations help with better curriculum vitae and even better job opportunities. As proceduralists, we have a self-serving interest to discuss the benefit of procedures. There remains no way to capture it all. Another weakness is that some presentations are done by junior faculty or trainees. Some may say these individuals may not know all the COIs for all the authors and thus our observations are not relevant. In fact, we feel that this is even more important. First, the more senior authors help guide the research project and agenda. Their disclosures are much more important and relevant. Second, if a senior author is assisting with a project enough to have their name on the presentation, they should have reviewed the data as well as the presentation. And thus, they should have made sure that the appropriate COIs were disclosed. Third, we were able to ascertain COIs via the OPD system. There is no reason trainees cannot do the same especially since SAGES explains this to all presenters.

Additionally, OPD has been known to have errors in reporting. Payments may be attributed to the wrong doctor or not attributed at all. We feel that our overall data will not be affected by this. While the ACCME and SAGES policies are similar regarding disclosures, they are not the same. Our view is that disclosures should be disclosed not for the purpose of fulfilling a policy of an organization but rather so that the audience may be given full disclosure of potential COI. We do not have evidence that the amount of financial relationship impacts the level of bias (or potential bias); however, logic dictates that a financial relationship of a few thousands of dollars is not similar to that of a few hundred thousands of dollars. Even if there is no bias, disclosing the exact amount gives the audience full data.

## Conclusions

Disclosure allows audiences to decide if the relationships impact the data presented. Full disclosure is imperative to root out any conscious or subconscious bias. Social science infers that the size of the payments matters. This is something that has not been addressed by either ACCME, nor at the 2023 SAGES. In fact, most scientific research, presentations, and publications have yet to address the million-dollar elephant in the room. Disclosing the mere existence of a relationship with an ineligible company is insufficient. In light of such a substantially high dollar amount, it is imperative that the actual amount should be part of true COI disclosure. Even if the research is not directly related to a specific company, it should be left to the audience to determine what bias exists or does not exist. All presentations must start including the dollar amounts for full disclosure of potential COI for future programs, not just in SAGES but in all ACCME activities.
